# SARS-CoV-2: The Lombardy scenario in numbers

**DOI:** 10.1017/ice.2020.115

**Published:** 2020-04-07

**Authors:** Andrea Molinari, Federico Pistoia, Giuditta Antonelli

**Affiliations:** 1 Department of Health Sciences (DISSAL), University of Genova, Genoa, Italy; 2 University of Genova, Genoa, Italy

*To the Editor—*On March 11, 2020, the World Health Organization (WHO) declared the spread of severe acute respiratory syndrome coronavirus 2 (SARS-CoV-2) to constitute a pandemic of COVID-19 infectious disease.^1^ On February 20, 2020, the first national cluster in Italy was identified in the Lombardy region after the diagnosis of SARS-CoV-2 in a 38-year-old man with a severe pneumonia and no relevant exposure history.^2^ To date, 74,386 SARS-CoV-2 laboratory-confirmed cases have been reported in Italy, with 32,346 cases in Lombardy alone, by far the most affected region.^3^

Given the extent of the phenomenon, we must urgently consider how the rapid spread of the infection can overload the National Health Service (SSN) and affect the mortality rate. The SSN is regarded as a high-level healthcare service, and it is regionally based.^4^ Specifically, Lombardy’s healthcare service is considered a benchmark in terms of quality and efficiency.^5^

In Lombardy, region of ~10 million people, the pre-crisis total intensive care unit (ICU) bed capacity was of ~720 beds, with a mean occupancy rate in the winter months of 85%–90%.^2^ To deal with SARS-CoV-2 outbreak, the number of ICU beds has significantly increased, and several departments have been reorganized and dedicated exclusively to COVID-19 patients. Nonetheless, hospitals in Lombardy are dramatically overcrowded with lack of medications, mechanical ventilators, oxygen, and personal protective equipment (PPE).^6^ Clearly, the increased number of cases is posing a serious threat to the entire SSN.^7^

We believe that the following numbers regarding the Lombardy region help to fully measure and elucidate the medical and social impact of SARS-CoV-2 outbreak.

The Italian National Institute of Health (ISS) reported that 4,451 people died in Lombardy due to SARS-CoV-2 complications between January 3 and March 25, 2020.^3^ In March 2019, there were 9,062 deaths, with 292 deaths per day^8^; in March 2020, the number of deaths per day was exceeded for 8 days by the number of deaths of confirmed COVID-19 patients alone. The most deadly day was March 21, with 546 daily fatalities due to COVID-19.

To date, 11,262 COVID-19 patients have been hospitalized—1,236 in an ICU. More than 5,000 healthcare workers have been infected across Italy, accounting for 9% of total cases. This number particularly reflects the lack of PPE and the unexpected pressure on the SSN.^3^

This report highlights how the impact and the consequences of the COVID-19 pandemic have been largely underestimated in Western countries, and it raises concerns about the potential responsiveness of healthcare systems in less-developed countries.
